# Central auditory processing and word discrimination in patients with multiple sclerosis

**DOI:** 10.1007/s00405-013-2776-6

**Published:** 2013-10-23

**Authors:** Ayub Valadbeigi, Farzad Weisi, Nematolah Rohbakhsh, Mohammad Rezaei, Atta Heidari, Amir Rahmani Rasa

**Affiliations:** 1 Department of Audiology, Faculty of Rehabilitation Science, Hamadan University of Medical Sciences, Hamadan, Iran; 2 Department of Speech Therapy, Faculty of Rehabilitation Science, Hamadan University of Medical Sciences, Hamadan, Iran; 3 Department of Audiology, Faculty of Rehabilitation Science, Tehran University of Medical Sciences, Tehran, Iran; 4 Department of Occupational Therapy, Faculty of Rehabilitation Science, Hamadan University of Medical Sciences, Hamadan, Iran

**Keywords:** Central auditory processing, Duration pattern sequence test, Gaps in noise, Word discrimination test, Multiple sclerosis

## Abstract

Many multiple sclerosis (MS) patients with normal pure tone threshold suffer from difficulties in their hearing especially speech perception in background noise, which is possibly because of incompetence of central auditory processing in this group. Three audiologic tests including gap in noise test (GIN), duration pattern sequence test (DPST) and word discrimination score (WDS) were used for comparing a number of aspects of central auditory processing between patients with MS and normal subjects. Approximate threshold and percent of correct answers in GIN test, percent of correct answers in DPST test and monosyllabic discrimination in WDS test were obtained through cross-sectional non-invasive study conducted on 26 subjects with relapsing-remitting multiple sclerosis who had mean age of 28.9 (SD 4.1) years, and 26 18–40-year-old ones with normal hearing and mean age of 27.7 (SD 5.2). Results of this study demonstrate increased approximate threshold and reduction of percent of correct answers obtained from GIN test in patients with multiple sclerosis (*Pv* = 0.0001). Furthermore in patients with MS, the average of correct answers in DPST was lower than normal subjects and finally performance of MS subjects in WDS test in quiet environment was correlated with GIN threshold (*r* = −/624, *Pr* = /003). Results of the present study showed that patients with MS had defect in aspects of central auditory processing consisting of temporal resolution, auditory pattern and the memory for auditory task and difficulty in discrimination of speech in noisy environment that are related to the involvement of central nervous system.

## Introduction

Multiple sclerosis (MS) is a chronic inflammatory demyelinating disease of the central nervous system (CNS) that was first identified by “Jean Charcotin” [1, 2]. MS is a disease of unknown etiology which affects over two million people worldwide [3]. It is believed that an interplay between susceptibility genes and environmental factors contributes to the pathogenesis of MS [4]. Many MS patients with normal pure tone thresholds complain of difficulty in their hearing, especially speech perception in background noise [2]. Studies have reported abnormal auditory processing in subjects with MS such as problems with dichotic listening tasks and auditory temporal processing [5]. Few studies have shown that 40–55 % of people with MS have at least an experience of dysarthria or speech that is characterized by slowness, slurring, or difficulties in production or comprehension [6]. Speech is one of the most complex forms of pattern recognition and requires both spatial and temporal processing. As speech understanding problems in background noise are features of individuals with auditory processing problems and disorders of the central auditory nervous system, one might postulate that individuals with MS would also have this type of deficit. In fact, several studies have revealed that a high percentage (33–69 %) of individuals with MS experience difficulty in speech understanding when they are exposed to a competing stimulus [3, 7]. Hence, three audiologic tests including gap in noise test (GIN), duration pattern sequence test (DPST) and word discrimination score(WDS) were used for evaluating central auditory processing in two groups of normal subjects and ones with MS.

The GIN test was developed to provide a clinical tool for evaluating temporal resolution ability in a variety of cases particularly with central auditory disorders. Sensitivity and specificity of GIN test in lesions of central auditory system have been reported 72 and 94 %, respectively [8]. In a study conducted by Musiek and et al., the mean approximate gap detection thresholds for the GIN test were 4.9 ms for the right ear and 4.8 ms for the left one in 50 normal hearing listeners [9]. In contrast, the mean approximate gap detection thresholds for the GIN test were 8.5 ms for the right ear and 7.8 ms for the left one in 18 subjects with confirmed neurological involvement of the central auditory nervous system. This study demonstrated the clinical importance of the GIN in assessing temporal resolution function [7]. Another basic test that assesses auditory pattern perception is DPST which is sensitive in detecting cerebral and brainstem lesion and particularly impaired auditory cortex. Another study showed that the bilateral dorsolateral prefrontal cortex, parietal lobe, superior temporal gyrus (STG), thalamus, basal ganglia, left cingulate cortex, the right inferior and medial frontal areas are involved [3, 4]. Concerning differential lateralization effects of sound discrimination, it has been suggested that temporal aspects of acoustic perception are critical in determining hemispheric lateralization as well as being a basis for language and sound lateralization. Auditory areas of left hemisphere are proposed to subserve short acoustic transitions, whereas the corresponding auditory areas of right hemisphere are preferably process the longer time windows [5, 6].

This study was conducted upon the comparison of temporal resolution and duration pattern between MS and healthy 18–40-years-old participants. The main aim was to investigate the relationship between aspects of central auditory processing and word recognition skills in MS people and normal ones.

## Methods

### Participants

Two groups were evaluated: 26 subjects with MS ranging in age from 18 to 40 years and 26 normal subjects who were matched to MS group in age, gender and literacy. The MS participants were recruited from Iran Ms Institute. Inclusion criteria for randomly selected MS subjects based on their medical records, neurologist diagnosis and MRI examination were: (1) suffering from relapsing MS and (2) having an expanded disability status scale score (EDSS) less than 6 and for both groups these include (a) having no history of epilepsy, seizures and head injury, and, (b) having auditory thresholds lower than 20 dB HL at all frequencies evaluated (octave frequencies between 0.25 and 8 kHz), bilaterally. The control group was selected from siblings without any neurological or audiological problems that were matched in age, literacy and gender with MS group.

## Materials

### Temporal resolution testing: GIN test

A broad band noise with a 6 ms duration among which random number gaps are recorded, is applied. The test was conducted monaurally and randomly started in right or left ear for each subject. Subjects were asked to press the button as they felt the gap. If there was no gap, the subject response was considered false positive and when the button was pressed in but there was no response, an error would be recorded. While being confused when asked to count the number of intervals in the test, the subject was asked to count the spaces. Approximate threshold and percent of correct answers were obtained. The test contains a practice list and four test lists. Ten practice items preceded the administration of the test items to ensure that the subjects had understood the task. Each test list is composed of 0–3 silent intervals ranging from 2 to 20 ms embedded in 6-s segments of white noise. The location, number, and duration of the gaps-per-noise segment vary throughout the test for a total of 60 gaps that are presented in each of four lists. So, from clinical viewpoint, the test could be done via only two test lists instead of four, which reduces the administration time by half (approximately 16 min) [7].

### Duration pattern sequence test (DPST)

Patterns of this test are applied through three consecutive 1000 Hz tones, one of which has either of longer or shorter duration than the other two. The durations are either 500 ms (long) or 250 ms (short). Intertonal interval is 300 ms with the rise and fall times of 10 m. Six different combinations of long and short sequences are used (LLS,SLL, LSL, SSL, SLS, LSS). Each pattern is randomly presented 10 times for a total of 60 presentations. The subject is instructed to report the pattern perceived by saying the appropriate “long” and “short” perceptions, and to guess if the subject is not certain [7].

### Word discrimination score test (WDS)

Tape recorded materials were applied for all speech tests. Speech discrimination thresholds were determined by Persian version of monosyllabic words test that is assessed at 40 dB sensation level in quiet environment and white noise at 0 dB signal/noise (S/N) ratio [10]. The speech signal and noise were presented through a speech audiometer (Madsen OB 822). The opposite ear was masked for testing the bone-conduction, air-conduction pure tone and speech as needed. All tests were carried out safely through non-invasive stimulations after obtaining participant’s consent. Data analysis was done using independent *t* test with a confidence level of 95 % and Pierson test through SPSS version 16.

## Results

This study was conducted on 26 relapsing-remitting MS sufferers with mean age of 28.9 years (SD 4.1) as well as 26 18–40-years-old normal participants with mean age of 27.7 (SD 5.2) and normal hearing. Results are categorized based on the outcomes of three tests as follows:

### GIN results

**Fig. 1:**
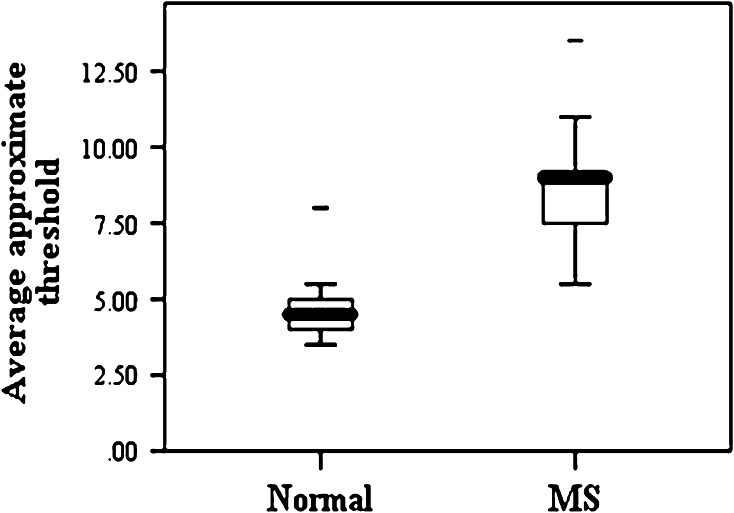
Mean and SD of approximate threshold in normal subjects and MS ones

**Fig. 2:**
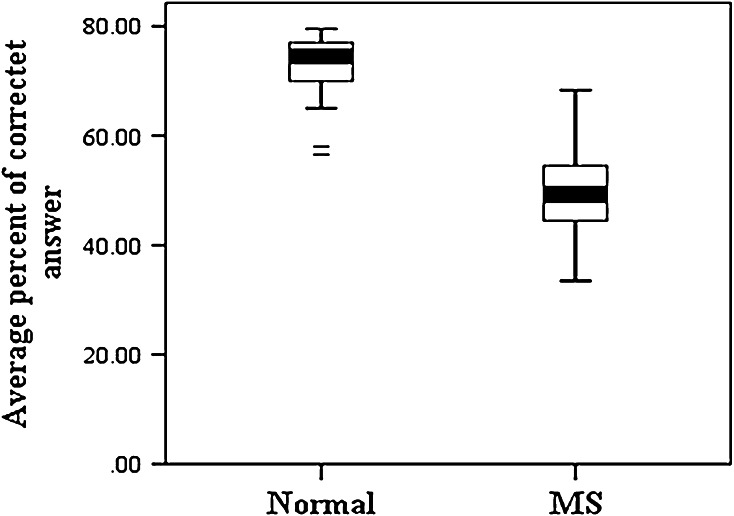
Mean and SD of average percent of correct answers in normal subjects and MS

Analysis of the approximate threshold and percent of correct answers including mean and standard deviation are shown in the following diagrams. There was no significant difference between the average approximate threshold (*Pv* = 0.68) and the percent of correct answers between men and women (*Pv* = 0.79). Furthermore, no significant difference was observed between the average approximate threshold (*Pv* = 0.67) and percent of correct answers in the case group (*Pv* = 0.40). But as shown in (Figs. 1, 2), significant difference appears between approximate threshold and percent of correct answers in normal subjects and patients (*Pv* = 0.001).

As shown above, approximate threshold and percent of correct answers are significantly different between normal subjects and patients (*Pv* = 0.001).

### DPST results

Frequency distribution of correct answers that is obtained from DPST including mean and standard deviation are shown in Table 1.

**Table 1:** Mean and standard deviation of DPST in normal subjects and patients with MS

	Normal (*n* = 26)				MS (*n* = 26)			
	Right ear		Left ear		Right ear		Left ear	
	Mean	SD	Mean	SD	Mean	SD	Mean	SD
DPST	85.6 %	6.5	86.4 %	6.1	64.3 %	6.9	67.6 %	5.6

	Male				Female			
	Right ear		Left ear		Right ear		Left ear	
	Mean	SD	Mean	SD	Mean	SD	Mean	SD
DPST								
Normal (*n* = 26)	85.7 %	6.7	87.6 %	6.5	85.4 %	66	85.3 %	5.8
MS (*n* = 26)	64.5 %	6.3	67.6 %	6.5	64.2 %	7.8	67.7 %	5.6

**Fig. 3:**
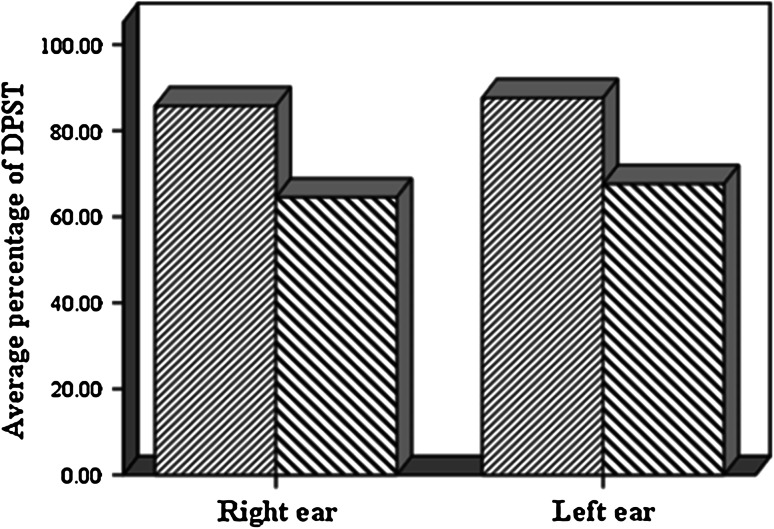
Comparison result of DPST in patients and normal women group

**Fig. 4:**
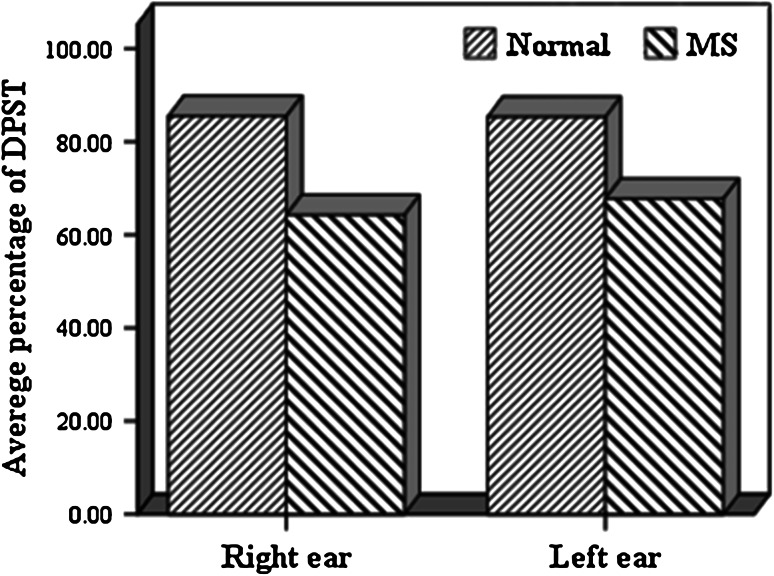
Comparison result of DPST in patients and normal men group

As noted in Table 1, there was no significance difference in the percentage of corrected answer of DPST between the right and left ears of normal subjects, however data analysis revealed that the percentage of correct answers from DPST in patient group had significant differences (*Pv* = 0.002). Percentage of correct answers of DPST in the right and the left ears in normal group also showed significant differences with MS group (Figs. 3, 4).

### WDS results

Results of the study in quiet and noisy environment include minimum and maximum scores, mean scores, and standard deviation shown in Table 2.

**Table 2:** Results of WDS in normal subjects and patients with MS

	Status	Min	Max	Mean	SD
Word discrimination score (%)					
Normal (*n* = 26)	Quiet	88	100	94.15	3.87
	Noise	54	92	71.07	11.10
MS (*n* = 26)	Quiet	88	100	94.30	3.57
	Noise	32	90	69.10	18.80

The performance of the MS group was similar to the normal one in white noise but with lower score than normal group, while both groups had normal hearing sensitivity and speech discrimination in quiet environment.

The subtle relationship between average threshold in GIN test and WDS revealed that by decreasing average threshold in GIN, WDS in quiet environment enhances in patient group (*Pv* = 0.003, *r* = −0.624). However, this relationship does not exist in noisy environment in patient group and quiet environment in normal group.

## Discussion

In this study, the approximate threshold and percent of correct answers of GIN test between normal subjects and ones MS were compared. Results showed no significant difference in the right and left ear between normal subjects and MS patients. No significant difference between the right and left ears was observed between the two groups. In some audiologic methods (including assessment of speech in noise) the right or tested ear is expected to be dominant related to left hemisphere dominance, however this pattern was not observed in this study which is compatible with previous studies of Brown and Nicholls [11] and Samelli [9]. The effect of gender on test results indicated no correlation between men and women in normal subjects and patients with MS on the approximate threshold and percent of corrected answers of GIN test that is similar to the study of Lotze, Snell, Hall and Grose, and phillips which reported no difference between men and women in the GIN test results [12–14]. On the contrary, in the study of Zaidan et al. [15] comparing GIN and (random gap detection test) RGDT in normal adults, sexual interest in both tests was shown to be higher in men. Another study in 2000 showed that women respond better than men on gap detection in more difficult auditory tasks (distance detection) so, women’s reaction time is faster due to their shorter gap detection [16].

According to results of the GIN test, it became obvious that in patient with MS, temporal resolution performance was poorer than the healthy group. In 2005, GIN test was used in 50 normal people and 18 patients with significant lesions in central auditory processing system, results indicated that the average approximate threshold in the right ear was 8.5 ms and 7.8 for the left ear that showed weaker performance of temporal resolution in people with auditory processing disorder. Given the overlap of the two results it can be mentioned that central auditory processing in people with MS is impaired [7].

Another study performed by GIN test on 44 subjects with normal hearing (less than dB HL 25) with and without tinnitus showed that those without tinnitus had shorter intervals than the ones with tinnitus. Actually, people with tinnitus had worse detection thresholds. Findings showed that even in people with tinnitus and normal hearing, tinnitus is likely to be caused by the defect or lack of afferent information and this confirms that damage to the cochlea leads to a series of changes in central auditory system as observed in tinnitus [17]. Moreover, results of this study indicate that DPST is sensitive to detect the cerebral lesion while it is not affected by mild to moderate hearing loss because of the fact that no frequency discrimination is required. Hence, only one frequency in supra threshold is used [18]. DPST may be more sensitive to cerebral lesion than the other central auditory processing tests using this paradigm such as pitch pattern. Absolutely, an advantage of DPST is its good sensitivity and specificity at least for cerebral and cochlear dysfunction. It also can be used to assess children with impaired language skills.

Patient with cerebral dysfunction demonstrated no problem with word discrimination in quiet environment; performance of DPST in quiet environment was poorer than WDS, showing that this pattern had more complexity since it required nonlinguistic and linguistic processing. Thus DPST might be more applicable to diagnose word discrimination. According to above-mentioned statements, poor performance of DPST in patients with MS demonstrated defect in central auditory processing such as auditory pattering and ordering memory.

In this study another variable was WDS, evaluating the effect of MS disorder on speech discrimination skills. Patients with MS showed reduction in word discrimination in white noise in spite of normal hearing sensitivity for all audiometric test frequencies and excellent speech discrimination in quiet environment which is similar to the study of Morales–Garcia and Poole [19].

Results showed the variability of WDS in noisy environment of MS patients comparing with the quiet one, in fact, word discrimination enhancement in quiet environment was observed in this group. As the average threshold in GIN test increases, WDS decreases implying that decline in the environment noise can lead to the rise of WDS and eventually improvement of comprehension speech. MS is a disease that involves anywhere in the central nervous system such as pathways of auditory system and can affect the integrity of the auditory nerve. There are several studies related to anatomical location of timing in the basal ganglia, sensory and motor cortex, the cerebellum and the higher levels of cortex [20–22].

The prior studies estimated that between 55–40 % of people with MS have disorders such as dyslexia, speech with low speed, vague and difficult speech production and understanding [6, 23, 24]. Thus, it can be assumed that the central processing system, especially in temporal resolution and ordering pattern and word discrimination might be impaired. This could be considered as a reason for such speech disorders in the afore-mentioned population.

## Conclusion

Many MS patients with normal pure tone thresholds complain of difficulty in their hearing especially speech perception in background noise. Standard audiologic tests have focused on disorders of peripheral system and do not show the precise dysfunction of the central system. Some fundamental audiologic tests including GIN, DPST and WDS were used in this study. Results showed that patients with MS have defect in some aspects of central auditory processing (CAP) including temporal resolution, auditory pattern and memory for auditory task as well as difficulty in speech discrimination in noisy environment that may be related to the involvement of the central nervous system. Therefore, these tests along with other behavioral and electrophysiological ones can be used for monitoring the effectiveness of medication, rehabilitation and related therapies.

## Limitations

Finding MS patients with normal hearing without middle ear pathologies and persuading them to participate were one of the limitations. Patient’s fatigue could possibly influence results while testing, so they were allowed to rest enough for completing the test.
